# AMP-activated protein kinase regulates the expression of human telomerase reverse transcriptase

**DOI:** 10.1371/journal.pone.0207864

**Published:** 2018-11-26

**Authors:** Daum Jo, Rackhyun Park, Hyunju Kim, Minsu Jang, Eun-Ju Lee, Ik-Soon Jang, Junsoo Park

**Affiliations:** 1 Division of Biological Science and Technology, Yonsei University, Wonju, Republic of Korea; 2 Department of Obstetrics and Gynecology, Chung-Ang University School of Medicine, Seoul, Republic of Korea; 3 Division of Bioconvergence Analysis, Korea Basic Science Institute, Daejeon, Republic of Korea; University of Newcastle, UNITED KINGDOM

## Abstract

The expression of hTERT in tumor cells contributes to oncogenic transformation by promoting immortalization. For this reason, hTERT is one of the major targets for cancer therapy, and an efficient method to downregulate hTERT expression is required for treatment of hTERT-positive cancer. In this report, we demonstrated that inhibition of AMP-activated protein kinase (AMPK) downregulates the expression of hTERT. We screened cell signaling pathways in AMPK α1 knockout cells and found that AMPKα1 is required for activity of the hTERT promoter. AMPKα1 knockout cells showed decreased expression of hTERT mRNA and protein. We also demonstrated that compound C, a reversible AMPK inhibitor, suppressed the expression of hTERT. However, AMPK activators, including AICAR and metformin, did not increase the level of hTERT protein. Finally, we showed that tumor cells stably expressing hTERT are resistant to compound C treatment. These results indicate that AMPK activity is required for tumor progression.

## Introduction

The telomeres at each end of a chromosome become progressively shorter during the replication of normal cells, and shortened telomeres can induce cellular senescence and block tumor progression. Human telomeres contain many copies of TTAGGG nucleotide repeats as well as an associated protein complex [1, 2]. To maintain continuous cell proliferation and prevent cellular senescence, telomerase is expressed in many immortal cells such germline cells, embryonic stem cells, and tumor cells [3, 4]. Moreover, telomerase plays a role in apoptosis and DNA repair, thus contributing to cell survival [5, 6]. Consequently, telomerase is an attractive therapeutic target in human cancer [7]. Telomerase is composed of an RNA component, telomerase RNA (TR), and a protein component, telomerase reverse transcriptase (TERT) [8]. Since human TR (hTR) is present in many human cell types, human TERT (hTERT) appears to be the limiting component for telomerase activity [9–11]. In studies of hTERT regulation, the hTERT promoter was cloned and found to be specifically activated in human cancers [12, 13].

AMP-activated protein kinase (AMPK) is one of the crucial regulators of cellular metabolism in eukaryotes and also regulates cell growth, autophagy, and cell polarity [14]. AMPK is a sensor protein that detects the cellular energy status and is activated when the cellular ATP level is low [15, 16]. The AMPK complex is composed of catalytic α subunits and regulatory β and γ subunits, and multiple AMPK subunit isoforms (α1, α2, β1, β2, γ1, γ2, γ3) exist in mammals [17]. While AMPKα1 expression is ubiquitous, the expression of AMPKα2 is specifically high in muscle, heart, and liver [18, 19]. Several reports suggest that AMPK activation prevents cellular senescence and ageing [20]. AMPK is responsible for the protection of cells from cellular stress, and the responsiveness of AMPK signaling clearly declines with ageing. Therefore, loss of the AMPK response to cellular stress ultimately contributes to the ageing process [21, 22]. A recent study also showed that resveratrol treatment prevents cellular senescence by activating AMPK signaling [23]. However, the relationship between AMPK and cellular senescence/ageing is not fully understood.

The role of AMPK in cancer is more intriguing. Many studies have indicated that AMPK activation strongly inhibits cell proliferation in tumor cells via cell cycle modulators such as the p53-p21 axis [24–26]. Moreover, AMPK is a downstream component of LKB1 tumor suppressor signaling and an upstream component of the SC1/2/mTOR pathway [27]. AMPK-deficient cells are resistant to oncogenic transformation and tumorigenesis, suggesting that AMPK is a conditional oncogene [28, 29]. For this reason, the detailed relationship between AMPK and tumorigenesis should be studied further.

In this study, we screened AMPK-dependent transcription using AMPKα1 knockout cell lines and found that hTERT expression is dependent on the presence of AMPKα1. In addition, we demonstrated that the AMPK inhibitor compound C could block cancer progression by suppressing hTERT expression. Our results suggest that AMPK is a potential target for cancer therapy.

## Materials and methods

### Cell culture and reporter assay

HEK293T, H1299, A549 and IMR-90 cells were obtained from the American Type Culture Collection (ATCC). All cell lines were grown in DMEM medium (Welgene, Korea) supplemented with 10% fetal bovine serum (Gibco, Waltham, MA, USA) and 1% antibiotic-antimycotic solution (Welgene, Seoul, Korea). Generation of the AMPK α1 knockout cell lines with CRISPR/Cas9 was described previously [30]. For the reporter assay, cells were seeded in a 24-well plate in DMEM 12 h before transfection. The total amount of DNA used for the transfection was typically 0.5 μg per well, and each assay was normalized with Renilla luciferase. The dual luciferase reporter assay kit was purchased from Promega (Madison, WI, USA). hTERT-Luc plasmid contains 1.7-kb DNA fragment encompassing the hTERT gene promoter [31]. AMPKα1 and AMPK α1 kinase dead (K47R) plasmids were purchased from Addgene (Cambridge, MA, USA)

### Western blotting

For western blot analysis, polypeptides in whole cell lysates were resolved by SDS-PAGE and transferred to PVDF membrane filters. Proteins were detected with a 1:1000 or 1:5000 dilution of primary antibody using an enhanced chemiluminescence (ECL) system. Images were acquired using the LAS4000 system (GE Healthcare, Uppsala, Sweden) and Chemidoc-it 410 imaging system (UVP, Upland, CA, USA). The following primary antibodies were used: anti-telomerase (Abcam, Cambridge, UK, ab32020), anti-AMPKα1 (Cell Signaling Technology, Beverly, MA, USA, #2532), anti-acetyl-CoA carboxylase (ACC) (Cell Signaling Technology, #3676), anti-phospho acetyl-CoA carboxylase (p-ACC) (Cell Signaling Technology, #3661), anti-c-Myc (Santa Cruz Biotechnology, Santa Cruz, CA, USA, sc788) and anti-actin (ABM, Richmond, BC, Canada, G043).

### Quantitative real-time PCR

Total RNA from each sample was extracted using Trizol reagent (Invitrogen, Carlsbad, CA, USA, #15596), Reverse transcription was carried out with an M-MLV RT kit (Enzynomics, Daejeon, South Korea) according to the manufacturer’s protocol. The following primers were used for amplification: hTERT, forward (AGC ACC GTC TGC GTG AG) and reverse (CAG CTC GAC GAC GTA CAC AC); RPL4, forward (GCT CTG GCC AGG GTG CTT TTG) and reverse (ATG GCG TAT CGT TTT TGG GTT GT). Real-time PCR was performed with a Step One Plus Real-Time PCR system (ABI, Foster City, CA).

### Telomerase Repeated Amplification Protocol (Trap) assay

Telomerase activity was measured by the TRAP assay [32]. Cells were lysed with NP-40 lysis buffer (10 mM Tris-HCl pH 8.0, 1 mM MgCl_2_, 1 mM EDTA, 1% NP-40, 0.25 mM sodium deoxycholate, 10% glycerol, 150 mM NaCl, and 5 mM β-mercaptoethanol) containing a protease inhibitor cocktail on ice for 30 min. After centrifugation at 13,200 rpm for 20 min, TRAP reactions were performed with 0.5 μg of protein extract per reaction and incubation at 25°C for 30 min for telomere extension. The extended products were amplified by PCR using TS (5'- AAT CCG TCG AGC AGA GTT -3') and ACX (5'- GCG CGG CTT ACC CTT ACC CTT ACC CTA ACC -3') primers for 27 cycles of denaturation at 95°C for 30 s, annealing at 52°C for 30 s, and extension at 72°C for 45 s. NT (5'-ATC GCT TCT CGG CCT TTT-3') and TSNT (5'-AAT CCG TCG AGC AGA GTT AAA AGG CCG AGA AGC GAT-3') primers were added as an internal control. The PCR products were separated by 12% non-denaturing PAGE in 0.5× TBE. After electrophoresis, the gels were fixed with a solution containing 0.5 M NaCl, 50% ethanol, and 40 mM sodium acetate for 15 min.

### Clonogenic assay

H1299 cells were plated in six-well plates and transfected with plasmid encoding 3×HA-hTERT. Two days later, the transfected cells were selected for 20 days under 1 mg/ml G418 selection. Single colonies expressing 3×HA-hTERT were isolated and treated with compound C (1 μM/ml) for 10 days. At the end of treatment, the cells were fixed with methanol and stained with crystal violet, and the number of deeply stained colonies was counted for further analysis. The plasmid 3×HA-hTERT was purchased from Addgene (Cambridge, MA, USA).

### Statistical analysis

The results of the western blot, reporter assay and TRAP assay analysis were evaluated by a 2-tailed *t* test using Excel software (Microsoft, Seattle, WA, USA). *P* < 0.05 was considered significant.

## Results

### Signaling pathway screening of AMPKα1 knockout (KO) cells with promoter reporter constructs

**Fig 1:**
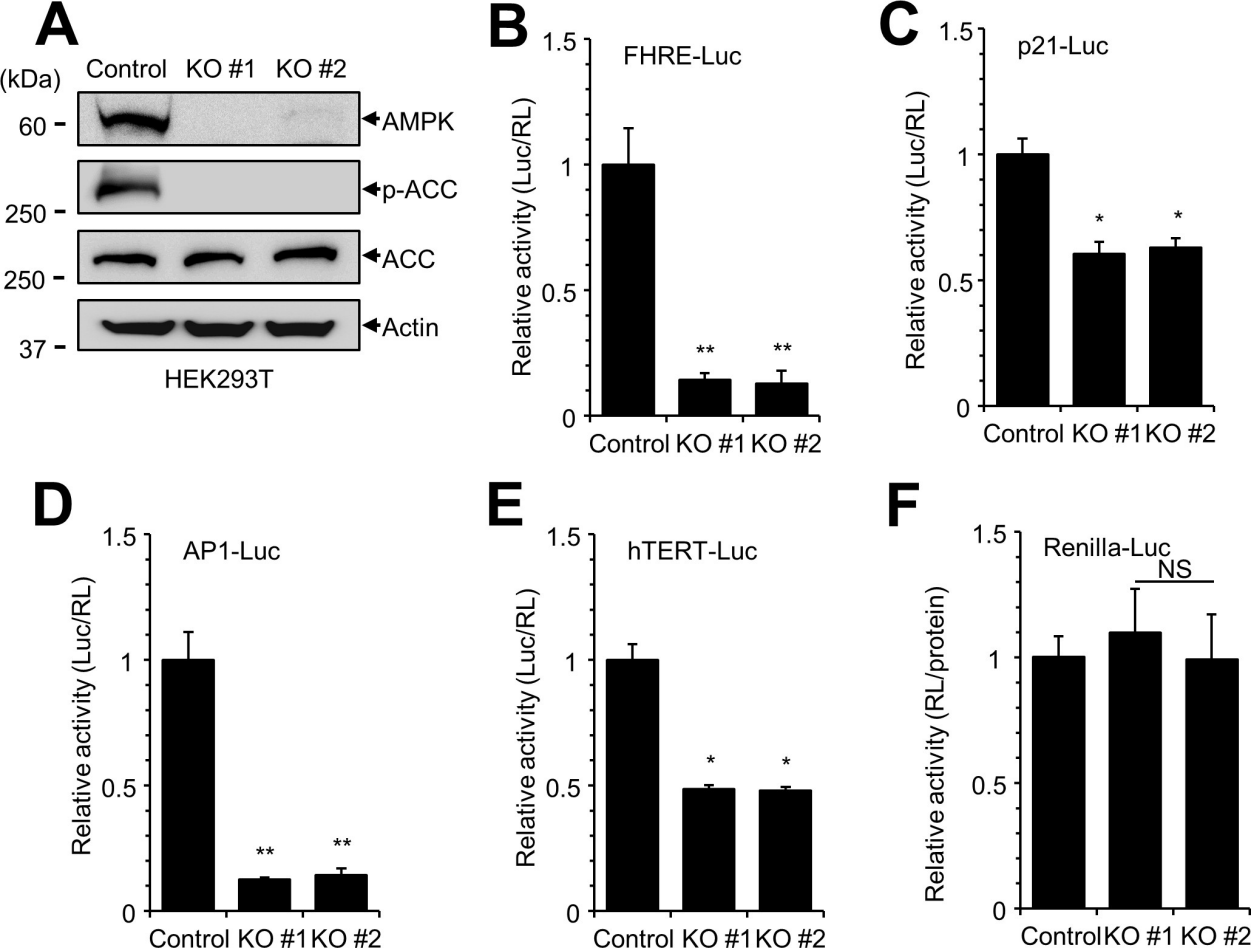
Signaling pathway screening in AMPKα1 knockout (KO) HEK293T cells. (**A**) The expression of AMPKα1 protein was examined in AMPKα1 knockout (KO) HEK293T cells by western blotting with the indicated antibodies. (**B-E**) HEK293 cells were transfected with various luciferase constructs (FHRE-Luc, p21-Luc, AP1-Luc, and hTERT-Luc) and internal control plasmids (pCMV-RL). At 24 h after transfection, luciferase reporter activity was measured and normalized to activity of Renilla luciferase. The experiment was performed in at least triplicate, and the graph shows average and standard error. Control vs. AMPKα1 KO, *P < 0.005, **P <0.0001, NS, not significant. (**F**) Plasmid encoding Renilla-Luc under control of the CMV promoter was used an as internal control to monitor the transfection efficiency. Renilla luciferase activity was normalized to total protein concentration in each sample.

To examine the signaling pathways of AMPK, we generated AMPKα1 knockout (KO) cell lines using the CRISPR-Cas9 gene editing system [30]. We used HEK293T cells because of their high transfection efficiency. We first confirmed that the expression of AMPKα1 was abolished in AMPKα1 KO cells, and that phosphorylation of adenylate cyclase, a substrate of AMPK, was decreased in these cells (Fig 1A). Next, we screened the cell signaling pathways modulated by AMPKα1 KO. AMPKα1 KO cells were transfected with luciferase constructs containing various promoters as follows: FHRE-Luc for FOXO3a-dependent transcription, p21-Luc for p53 signaling, AP1-Luc for MAP kinase signaling, and hTERT-Luc for telomerase activity (Fig 1B–1E). The relationship between the first three reporter constructs and AMPK was previously reported [24, 33, 34]. Interestingly, hTERT-Luc also showed a significant decrease in reporter activity (Fig 1E). However, the activity of renilla-Luc reporter containing CMV promoter was not decreased in AMPK α1 KO (Fig 1F). These data from signaling pathway screening suggest that AMPKα1 knockout affects telomerase activity.

### The level of hTERT expression is reduced in AMPKα1 KO cells

**Fig 2:**
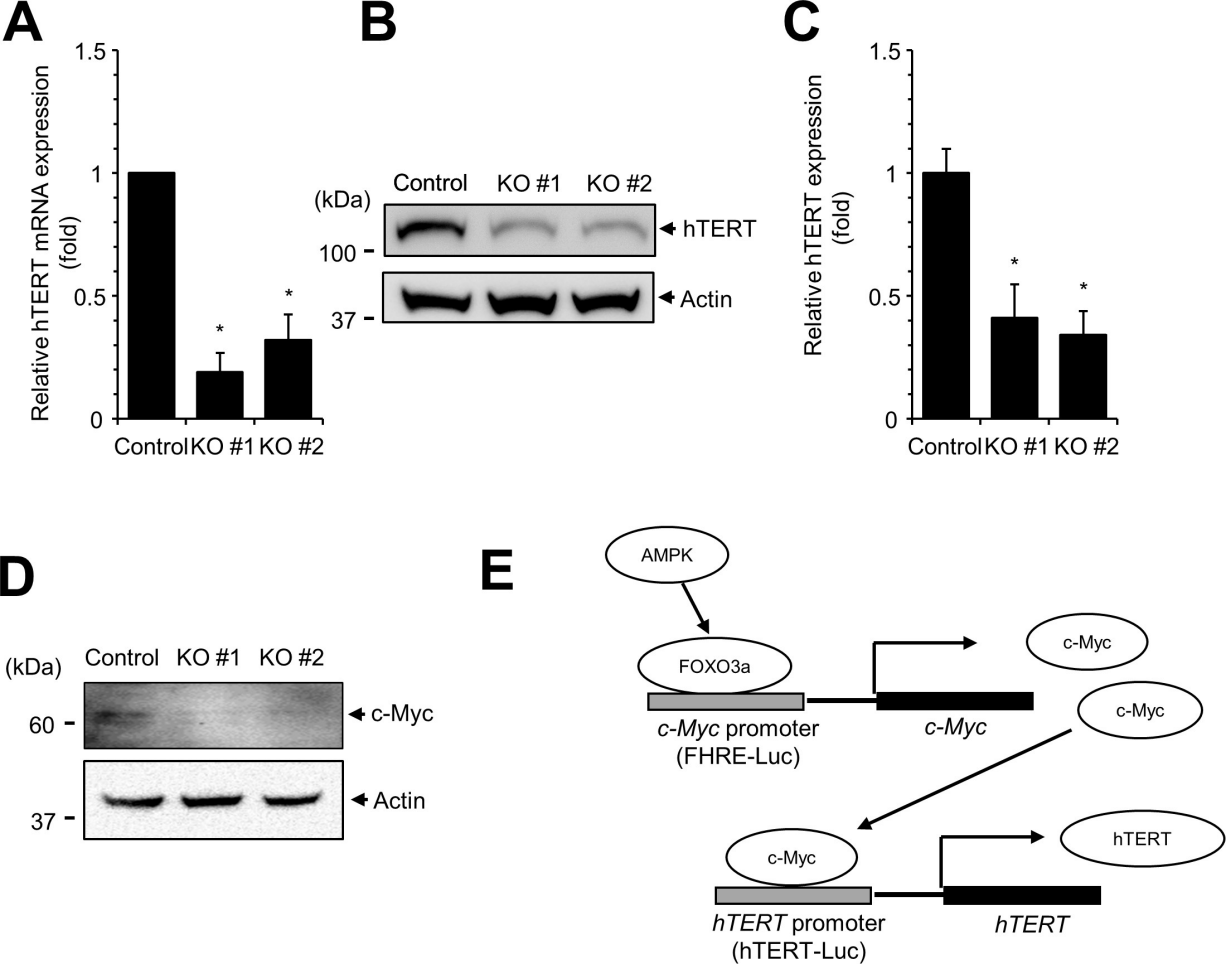
The level of hTERT expression is decreased in AMPKα1 KO cells. (**A**) The expression of hTERT mRNA was measured by quantitative real-time PCR in AMPKα1 KO cells. (**B**) The level of hTERT protein is decreased in AMPKα1 KO cells. Cell lysates were subjected to western blotting with anti-hTERT antibody. (**C**) The levels of hTERT protein were quantitated. Experiments were performed in triplicate, and the mean and standard deviations are shown in the graph. Control vs. AMPKα1 KO, *P < 0.005. (**D**) The expression of c-Myc protein was downregulated in AMPKα1 KO cells. (**E**) Schematic representation of AMPK induced activation of hTERT expression.

Because the activity of hTERT-Luc was decreased in AMPKα1 KO cells, we examined hTERT mRNA and protein expression in these cells. Quantitative RT-PCR and western blotting analyses showed that the expression levels of hTERT mRNA (Fig 2A) and hTERT protein (Fig 2B and 2C) were significantly decreased in AMPKα1 KO cells. These results collectively demonstrate that hTERT expression is decreased in AMPKα1 KO cells.

Because hTERT transcription was decreased in AMKα1 KO cells, we examined the expression of c-Myc protein. The expression of c-Myc is regulated by FOXO3a, and c-Myc also activates hTERT expression [35, 36]. Western blot analysis showed that c-Myc expression was decreased by AMPKα1 KO cells (Fig 2D). These results suggest that AMPK regulates hTERT expression via c-Myc (Fig 2E).

### AMPK inhibitors decrease hTERT promoter activity, but AMPK activators have no effect

**Fig 3:**
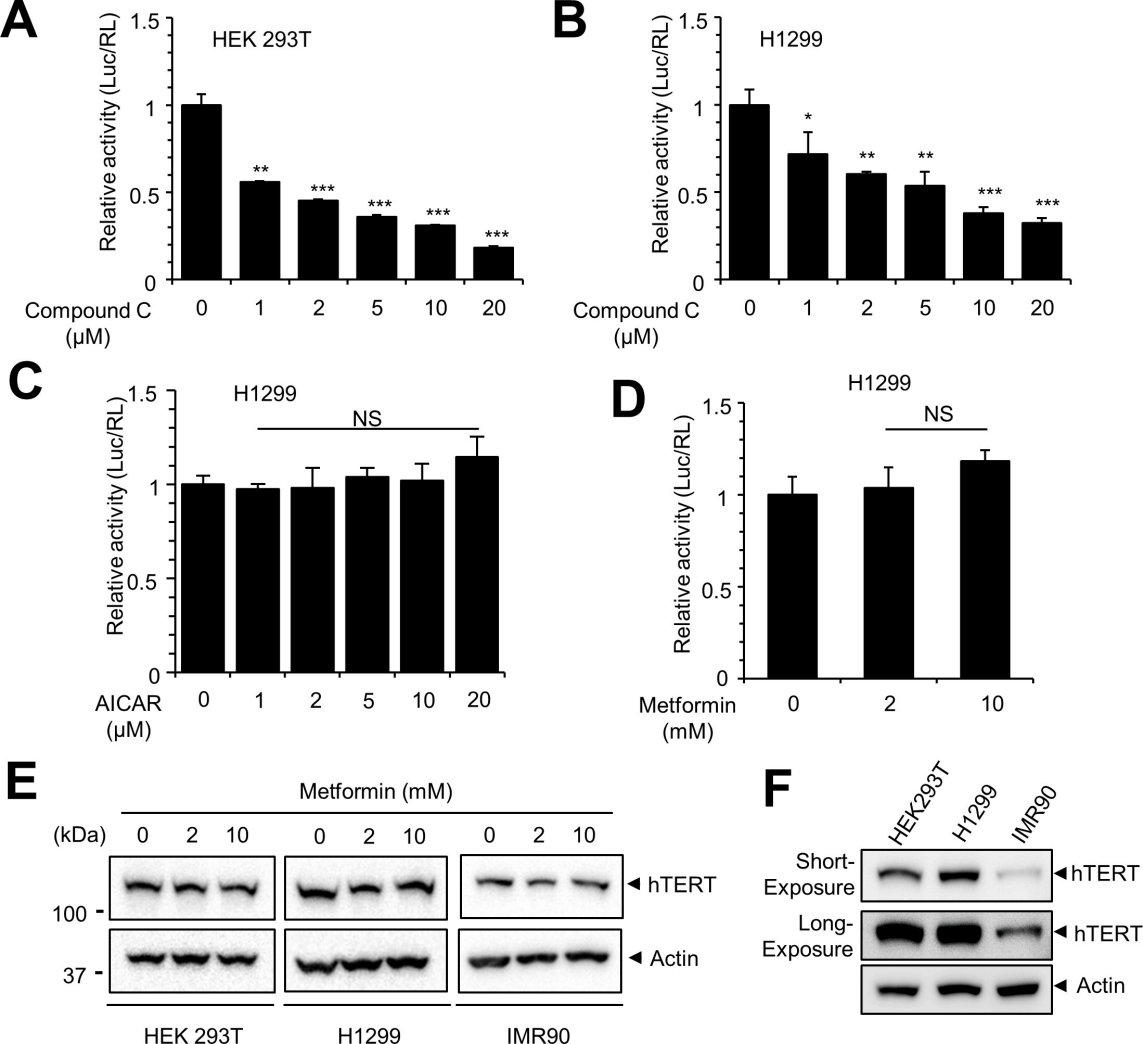
Compound C treatment decreases hTERT promoter activity, but AMPK activators do not increase the activity. (**A, B**) HEK293T and H1299 cells were transfected with hTERT-Luc plasmid. After 24 h after transfection, cells were treated with the indicated concentrations of compound C for 12 h, and the luciferase activity was measured. Relative luciferase activity was normalized to Renilla luciferase activity and is represented as a fold increase compared with the control. Control vs. drug treated, * *P* < 0.05, ** *P* <0.005 *** *P* < 0.001. (**C, D**) H1299 cells were transfected with hTERT-Luc plasmid. Cells were treated with the indicated concentrations of AICAR and metformin for 12 h, and the luciferase activity was measured. Relative luciferase activity was normalized to Renilla luciferase activity and is represented as a fold increase compared with the control. (**E**) HEK293T, H1299 and IMR90 cells were treated with the indicated concentration of metformin for 12 h, and the expression of hTERT protein was examined by Western blot. (**F**) Expression of endogenous hTERT protein in HEK293T, H1299 and IMR90. Equal amount of cell lysates were subjected to Western blot with the indicated antibodies.

A previous study demonstrated that compound C is an inhibitor of AMPK [37]. Because the activity of hTERT promoter was decreased in AMPKα1 KO cells, we examined whether compound C could pharmacologically repress the activity of hTERT promoter. HEK293T and H1299 cells were transfected with hTERT-Luc reporter construct, and the transfected cells were treated with compound C for 12 h. The luciferase activity of hTERT promoter in both HEK293T and H1299 cells was inhibited by compound C in a dose-dependent manner (Fig 3A and 3B). These results indicate that AMPK inhibition represses hTERT-Luc activity. Since AMPK inhibition decreases the activity of hTERT promoter, we examined the activity of the hTERT promoter after treatment with AICAR and metformin, activators of AMPK [38]. H1299 cells were transfected with hTERT-Luc reporter construct, and the transfected cells were treated with the indicated concentrations of AICAR and metformin for 12 h. The activity of hTERT promoter in H1299 cells was not significantly affected by treatment with either AICAR or metformin (Fig 3C and 3D). In addition, we examined hTERT expression with metformin treatment in HEK293T, H1299 and IMR90 (a fetal lung fibroblast) cells. Western blot data showed that the expression of hTERT protein was not affected by metformin, indicating that AMPK activation does not influence the activity of the hTERT promoter (Fig 3E). We detected relatively weak hTERT expression in IMR90 cells (Fig 3F). Since the expression of hTERT protein in IMR90 is supposed to be negative [39], the hTERT band in IMR90 could to be due to an artefact.

### AMPK inhibition interferes with hTERT expression

**Fig 4:**
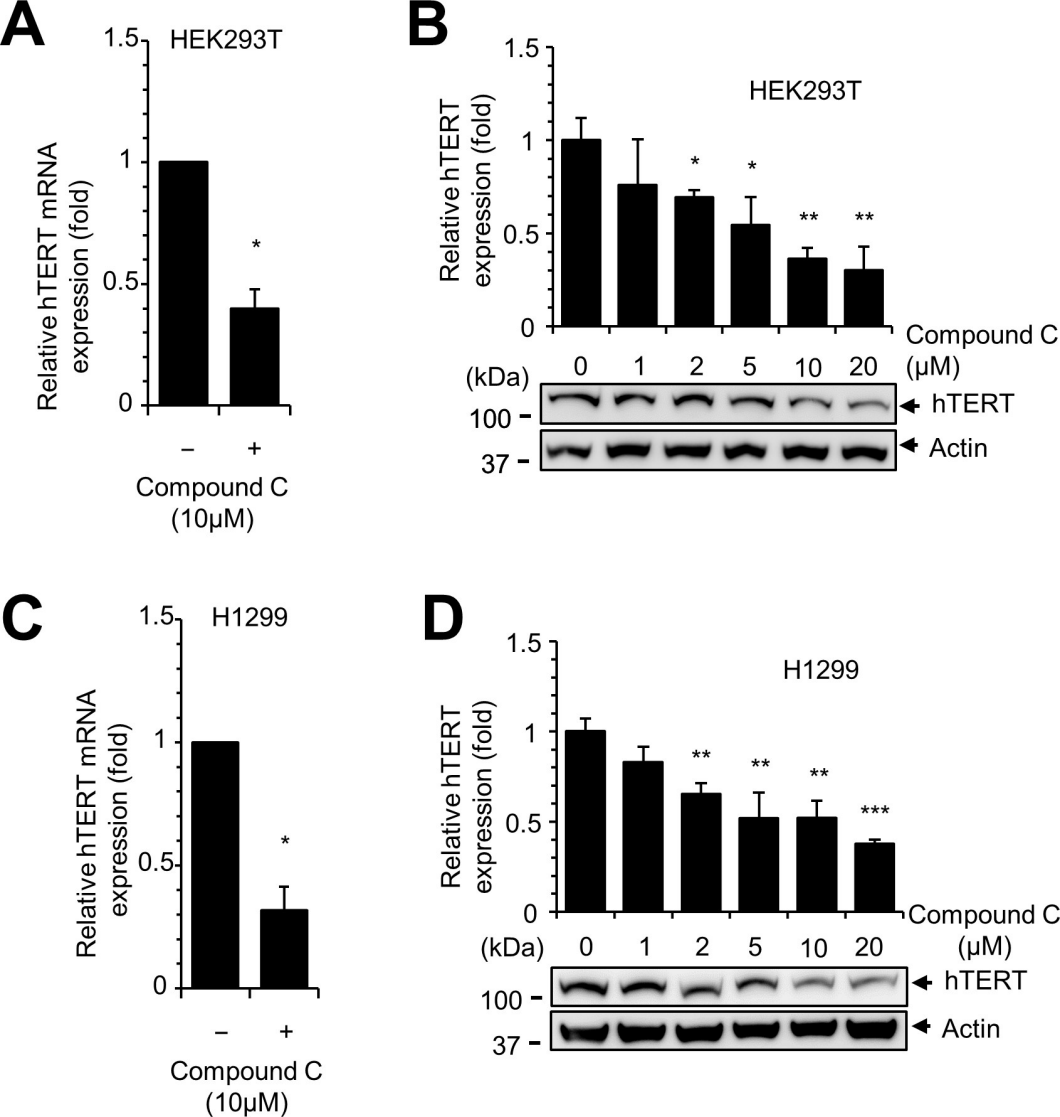
Compound C reduces hTERT expression. (**A, B**) Compound C treatment decreases expression of hTERT mRNA and protein. HEK293T cells were treated with compound C for 12 h, and the expression of hTERT was measured by quantitative real-time PCR and western blotting. Control vs. drug treated, * *P* < 0.05, ** *P* < 0.005, *** *P* < 0.001. (**C, D**) H1299 cells were treated with the indicated concentrations of compound C for 12 h, and the expression of hTERT was measured by quantitative real-time PCR and western blotting.

Since compound C decreased the activity of hTERT promoter, we hypothesized that hTERT expression is decreased by AMPK inhibition. To test our hypothesis, we treated HEK 293T and H1299 cells with compound C and examined the expression of hTERT mRNA and protein. Quantitative RT-PCR analysis revealed that the expression of hTERT mRNA was reduced by compound C treatment in these two cell lines (Fig 4A and 4C). Similarly, compound C decreased the level of hTERT protein in a dose-dependent manner (Fig 4B and 4D). These results demonstrate that compound C decreased hTERT expression.

**Fig 5:**
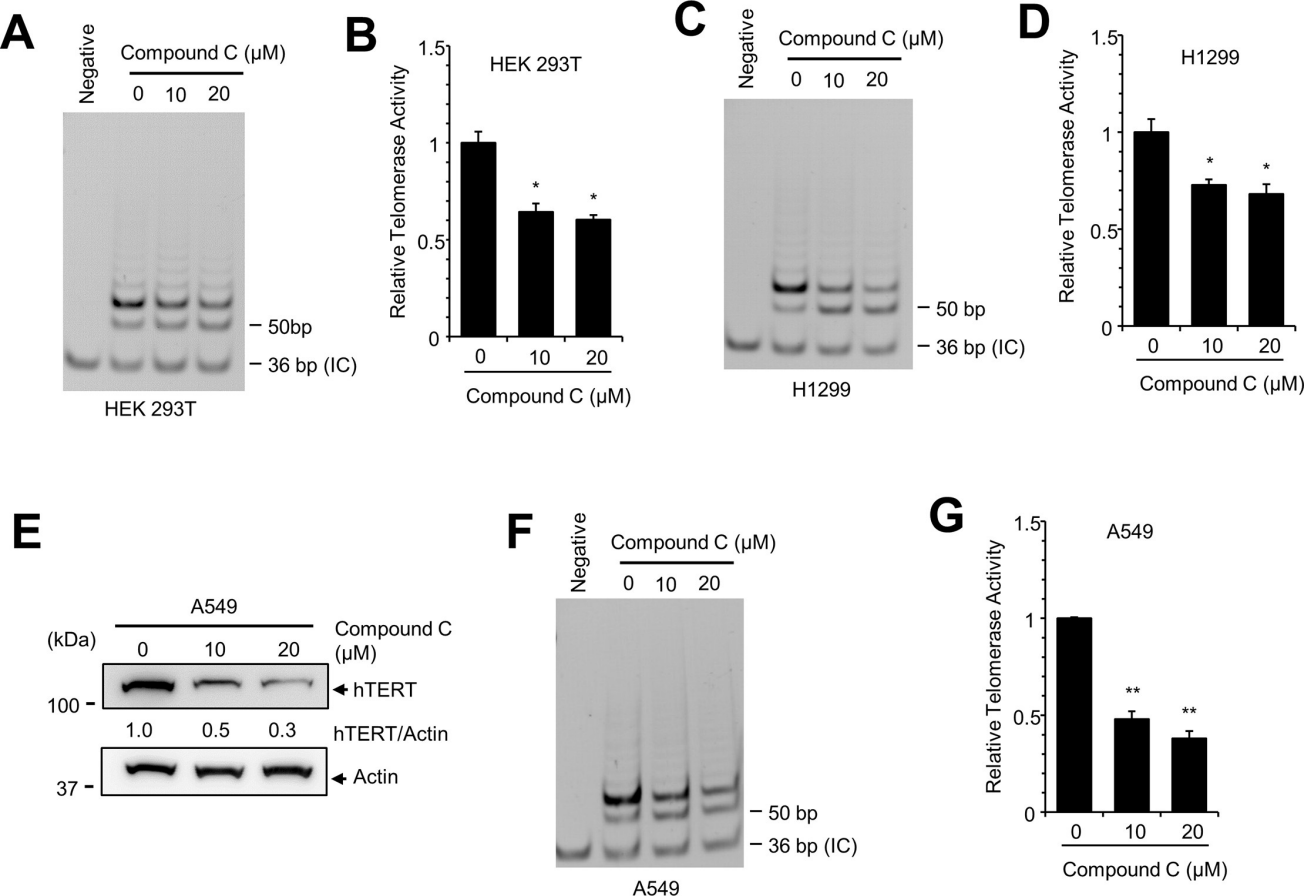
Compound C treatment reduces telomerase activity. (**A**) HEK293T cells were treated with indicated concentrations of compound C for 12 h. Cell lysates were prepared and assayed by TRAP analyses. The 36-bp band represents the internal TRAP assay standards (IC, internal control). (**B**) PCR-amplified extension products were quantified to measure the telomerase activity. (**C, D**) TRAP analysis of H1299 cells. (**E**) A549 cells were treated with the indicated concentrations of compound C for 12 h, and the expression of hTERT was examined by western blotting. The level of hTERT expression was quantitated and normalized to actin expression. (**F, G**) TRAP analysis of A549 cell lysates. Control vs. drug treated, * *P* < 0.05, ** *P* < 0.005.

Finally, we examined telomerase activity in HEK293T and H1299 cells using the telomerase repeated amplification protocol (TRAP) assay [32]. Although TRAP activity was not decreased by low concentrations of compound C (data not shown), high concentrations of compound C (10 μM and 20 μM) considerably decreased TRAP activity in HEK293T and H1299 cells (Fig 5A–5D). To confirm these results, we examined the effect of compound C in A549 lung tumor cells and showed that compound C decreased the expression of hTERT protein and also decreased telomerase activity in A549 cells (Fig 5E–5G). These results indicate that compound C represses hTERT expression as well as telomerase activity.

### Expression of AMPKα1 rescues hTERT expression

**Fig 6:**
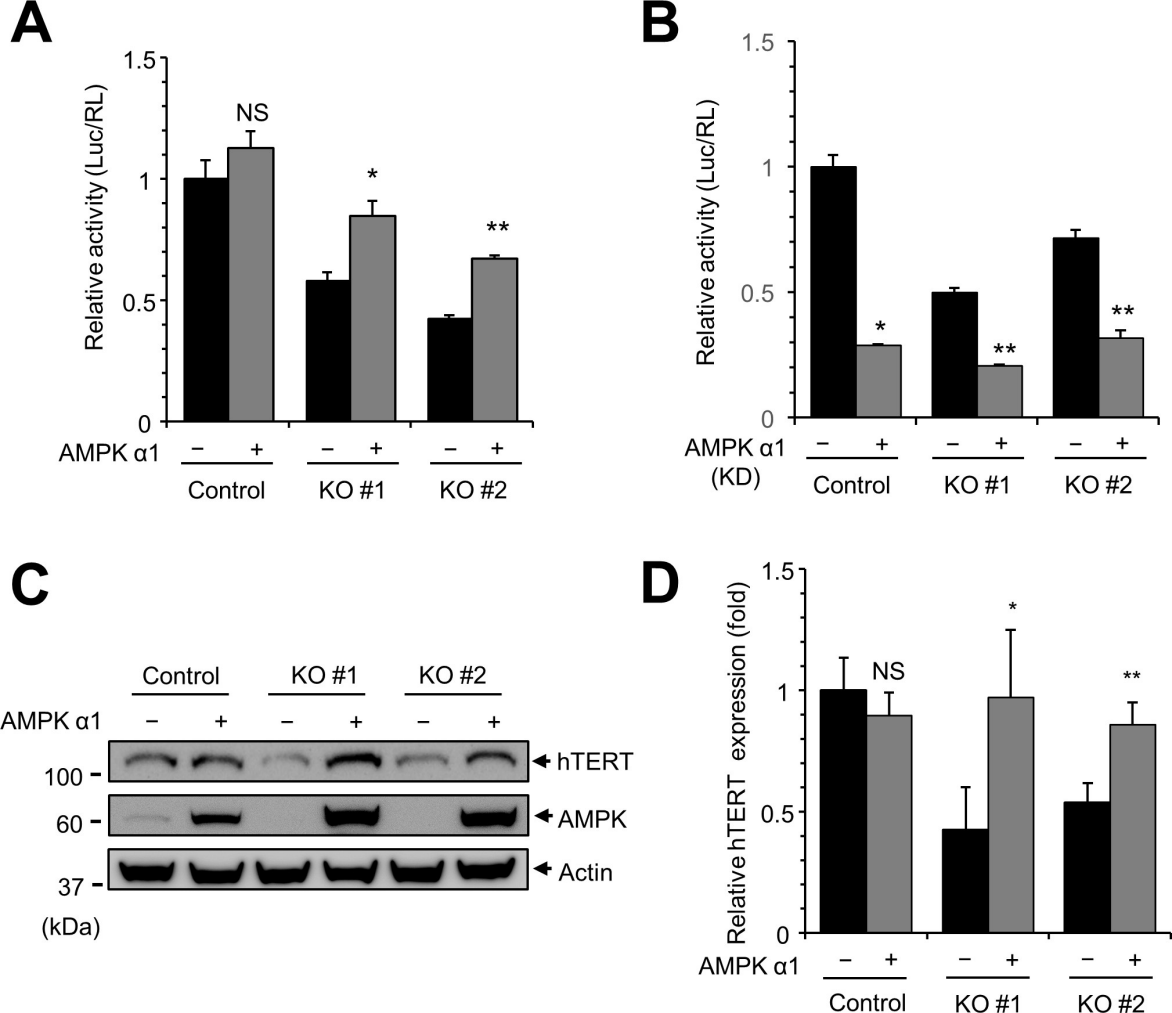
Transient expression of AMPKα1 rescues hTERT expression. (**A**) Transient expression of AMPKα1 rescues hTERT-Luc activity. Control cells and AMPKα1 KO cells were transfected with hTERT-Luc plasmid with either empty vector or plasmid encoding AMPK. At 24 h after transfection, luciferase reporter activity was measured. Luciferase activity was normalized to activity of Renilla luciferase. Empty vector vs. AMPKα1, * *P* < 0.05, ** *P* < 0.01, NS, not significant. (**B**) Transient expression of AMPKα1 kinase dead (KD) does not rescue hTERT-Luc activity. (**C**) Transient expression of AMPKα1 increases the level of hTERT protein in AMPKα1 KO cells. AMPKα1 KO cells were transfected with either empty vector or plasmid encoding AMPKα1. At 24 h after transfection, cell lysates were subjected to western blotting with anti-hTERT antibodies and anti-AMPK antibodies. (**D**) Quantification of hTERT and AMPK bands.

Because hTERT expression is reduced in AMPKα1 KO cells, we investigated whether overexpression of AMPKα1 can rescue the expression of hTERT. AMPKα1 knockout cells were transfected with plasmid encoding hTERT-Luc in the presence or absence of plasmid encoding AMPKα1. Restoration of AMPKα1 resulted in the recovery of hTERT promoter activity (Fig 6A). However, the expression of AMPKα1 kinase dead mutant (K47R) did not rescue the activity of hTERT promoter indicating that the kinase activity of AMPKα1 is required for hTERT expression (Fig 6B). In addition, transient expression of AMPKα1 increased the level of hTERT protein (Fig 6C and 6D). These results collectively indicate that AMPKα1 expression can rescue hTERT expression.

### Tumor cells stably expressing hTERT are more resistant to compound C treatment

**Fig 7:**
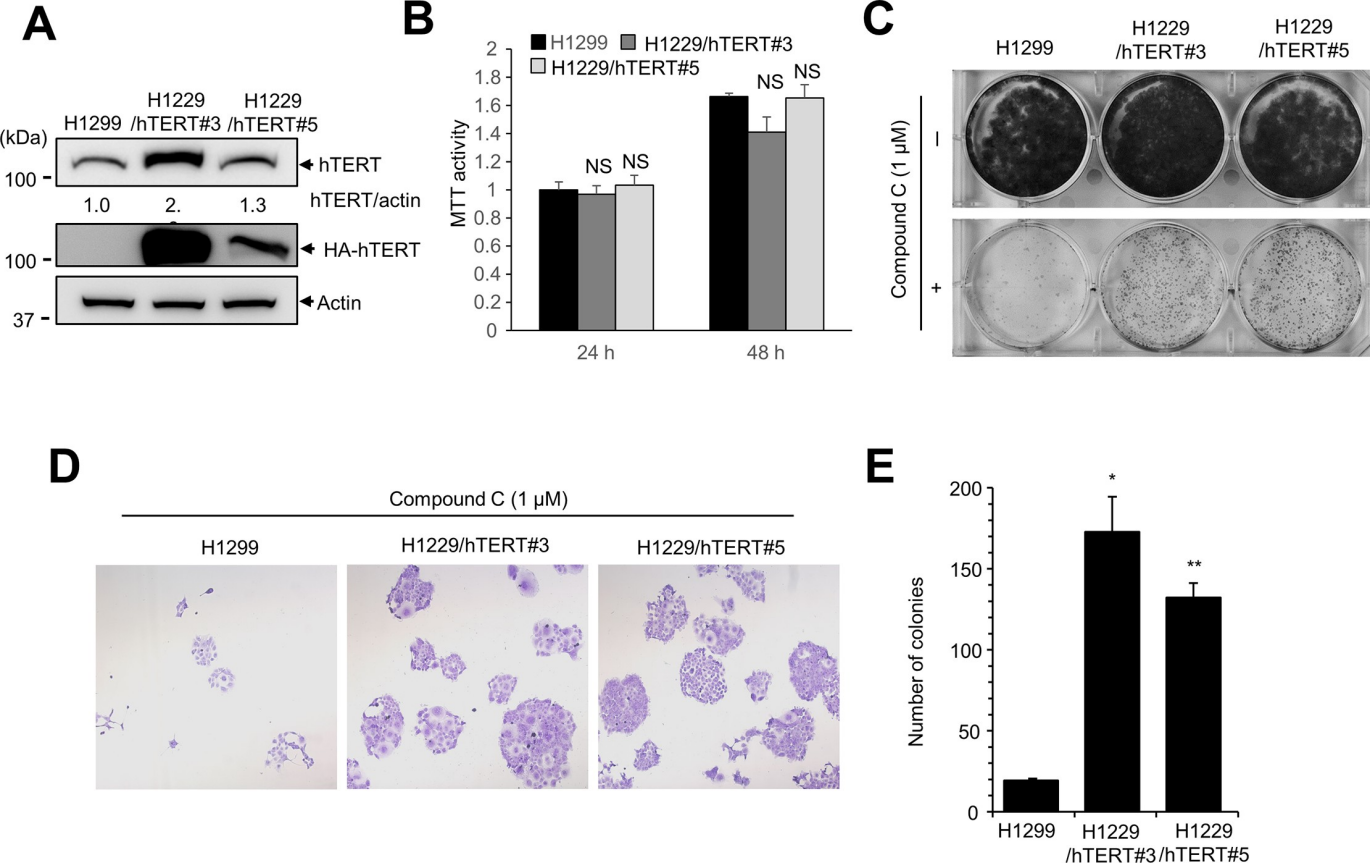
H1299 cells expressing hTERT are resistant to compound C treatment. (**A**) H1299 cells were transfected with plasmid encoding 3×HA-hTERT to generate H1299 cells stably expressing hTERT (H1299/hTERT). Western blotting demonstrated that HA-hTERT was expressed in H1299/hTERT cells. Equal amounts of H1299 control and H1299/hTERT were subjected to western blotting with anti-hTERT antibody and anti-HA antibody. (**B**) MTT assay showed no significant difference between H1299 and H1299/hTERT. Equal number of cells (8,000 cell/well) were seeded in 24 well plates, and MTT assay were performed at the indicated times. H1299 vs. H1299/hTERT. NS, not significant. (**C**) Clonogenic assays were carried out to assess the effects of hTERT overexpression on cell proliferation. H1299/hTERT cells were treated with compound C (1 μM) for 10 days before fixation and staining with crystal violet. (**D**) Light microscopy (200×) images of clonogenic assays. (**E**) The number of colonies was counted. H1299 vs. H1299/hTERT, * *P* <0.01, ** *P* <0.005.

Previous studies showed that compound C interferes with the proliferation of cancer cells [40]. Because hTERT expression is often upregulated in cancer cells, we examined whether overexpression of hTERT is associated with resistance to compound C. First, we generated H1299 cells stably expressing hTERT. H1299 cells were transfected with plasmid encoding HA-tagged hTERT, and hTERT-expressing colonies (H1299/hTERT) were isolated (Fig 7A). Cell proliferation assay showed no significant difference between control H1299 cells and H1299/hTERT (Fig 7B). Next, we assessed the effect of hTERT overexpression on resistance to compound C by clonogenic assay. H1299/hTERT cells were treated with compound C for 10 days. Whereas compound C treatment alone decreased the number and size of colonies, hTERT overexpression increased cell survival in the presence of compound C (Fig 7C). Observation of the colonies by light microscopy revealed that the colonies were larger and the number of colonies was significantly increased for H1299/hTERT cells compared with controls (Fig 7D and 7E). These results suggest that hTERT overexpression contributes to resistance to compound C.

## Discussion

In this study, we used AMPKα1 KO cell lines to screen cell signaling pathways. Our data confirmed that AMPK is involved in AP1-, p53-, and FOXO3a-dependent transcription, suggesting that the AMPKα1 KO system is suitable for screening of cell signaling [24, 33, 34]. During these experiments, we found that activity of the hTERT promoter (hTERT-Luc) was decreased in AMPKα1 KO cells and therefore studied the role of AMPK in hTERT expression. Studies using a Renilla-Luc reporter construct containing the CMV promoter (Fig 1F) revealed that activity of the CMV promoter was not affected by AMPKα1 KO, indicating that repression of hTERT promoter activity in AMPKα1 KO cells was not due to general transcription repression or a result of different transfection efficiency. A previous study revealed the detailed regulatory elements of hTERT promoter and demonstrated that hTERT promoter activity can be regulated by AP-1 and FOXO3a [35, 41]. We therefore speculate that AMPK regulates hTERT expression by modulating AP-1- and FOXO3a-dependent transcription (Fig 1). In addition, we examined the expression of c-Myc protein in AMPKα1 KO cells (Fig 2). The expression of c-Myc is regulated by FOXO3a, and FOXO3a dependent promoter (FHRE-Luc) was significantly reduced in AMPKα1 KO cells (Fig 1). Western blot analysis showed that the expression of c-Myc was reduced in AMPKα1 KO cells suggesting that hTERT expression is repressed in AMPKα1 KO cells via reduced c-Myc expression (Fig 2). However, AMPK may use additional molecules to regulate hTERT expression. Further study will be required to reveal the detailed link between hTERT expression and AMPK expression.

We confirmed our finding by demonstrating reduced expression of hTERT in AMPKα1 KO cells and in compound C-treated cells. We clearly showed that the expression of hTERT mRNA and protein was decreased by AMPK inhibition; however, AMPK activation did not result in an elevated level of hTERT activation. Treatment of H1299 cells with AICAR or metformin did not result in a significant increase in hTERT promoter activity (Fig 3C and 3D), and we did not detect a dramatic increase in hTERT promoter by AMPKα1 overexpression in control cells (Fig 6A). These results suggest that the appropriate expression of AMPKα1 is required for hTERT expression. In addition, previous reports showed that knockdown of AMPK in differentiated T cells activated the expression of hTERT suggesting that the relation between AMPK and hTERT is not simple [42]. The current data suggest that efficient hTERT expression requires AMPK activity; however, excessive activation of AMPKα1 does not result in overexpression of hTERT.

We also used a TRAP assay to measure the activity of hTERT. Treatment with compound C reduces the activity of telomerase by decreasing the expression of hTERT (Fig 5). We therefore expected to find reduced TRAP activity in AMPKα1 KO cells, but did not detect any significant decrease (data not shown). We speculate that compound C affected the expression of AMPKα1 in the short term, and the decreased expression of AMPKα1 affected the telomerase activity. However, AMPKα1 KO cells were maintained for a long time during selection and proliferation and adapted during this time such that the shortage of hTERT appears to be compensated by other methods such as increased activity of telomerase. When we maintained AMPKα1 KO HEK293T cells for a long time we did not observe any senescence-like phenotype with reduced expression of hTERT. Further study will be required to elucidate the relationship between hTERT expression and TRAP activity.

A previous study showed that compound C, an AMPK inhibitor, can block cancer cell proliferation by modulating p53-p21 signaling, FOXO3a, and autophagy. In this report, we demonstrated that elevated expression of hTERT can partially protect cancer cells from compound C (Fig 7). Comparison of the results of clonogenic assay between mock and compound C treatment suggested that elevated expression of hTERT did not completely protect the tumor cells from compound C; however, our results do indicate that elevated expression of hTERT provides resistance to compound C. Since AMPK activation by AICAR or metformin is one option for the treatment of cancer, the relationship between AMPK and hTERT expression should be considered in such cases.

In this report, we show reduced expression of hTERT in AMPKα1 KO cells and discuss the role of hTERT in telomerase activity. As many studies have shown that telomerase is involved not only in telomere maintenance, but also in other cellular process including apoptosis and DNA repair [5, 6], the reduced expression of hTERT by AMPKα1 could result in increased apoptosis and decreased DNA repair activity. Reduced expression of telomerase may therefore affect tumor cell survival and proliferation.
